# Imaging diagnosis of Caroli-like disease with concurrent extrahepatic biliary obstruction in a young small-breed dog: case report

**DOI:** 10.3389/fvets.2026.1717663

**Published:** 2026-05-29

**Authors:** Jinkyung Park, Sung-Yeon Cho, Matti Kiupel, Kichang Lee, Hakyoung Yoon

**Affiliations:** 1 Department of Veterinary Medical Imaging, College of Veterinary Medicine, Jeonbuk National University, Iksan, Republic of Korea; 2 Kokkiri Animal Medical Center, Jeonju-si, Jeollabuk-do, Republic of Korea; 3 Veterinary Diagnostic Laboratory, Michigan State University, Lansing, MI, United States; 4 Antech Asia, Kowloon, Hong Kong SAR, China; 5 Biosafety Research Institute and College of Veterinary Medicine, Jeonbuk National University, Iksan, Jeollabuk-do, Republic of Korea

**Keywords:** biliary tract disease, computed tomography, diagnostic imaging, ductal plate malformation, gallbladder duplication, hepatic fibrosis

## Abstract

Caroli-like disease is a rare congenital ductal plate malformation characterized by intrahepatic bile duct dilatation and has only rarely been reported in veterinary medicine. A 2-year-old castrated male small-breed dog (5.4 kg) was presented with jaundice, weight loss, and lethargy. Laboratory tests revealed elevated liver enzyme activities, hyperbilirubinemia, and coagulopathy. Radiography showed two mineralized opacities superimposed on the hepatic silhouette. Ultrasonography revealed cholecystolithiasis, gallbladder wall thickening, and biliary dilatation with choledocholithiasis, suggesting extrahepatic biliary obstruction. Computed tomography confirmed choledocholithiasis, diffuse biliary dilatation, and a 2- to 3-cm intrahepatic cystic structure containing calcified material and communicating with the intrahepatic bile ducts. Histopathological analysis revealed cholangiohepatitis with ductal proliferation, and immunohistochemical analysis confirmed the biliary epithelial origin of the lesion and supported a congenital ductal plate malformation. Despite surgical management, the patient died postoperatively. This case represents the first veterinary description of Caroli-like disease complicated by extrahepatic biliary obstruction in a young dog and highlights the fact that intrahepatic saccular dilatation may resemble gallbladder anomalies on ultrasonography, while computed tomography provides crucial diagnostic clarity. Recognition of such rare conditions may improve diagnostic accuracy and clinical decision-making in veterinary hepatobiliary disorders.

## 1 Introduction

Ductal plate malformations (DPMs) are developmental anomalies of the biliary tree, representing a continuum of fibrocystic liver diseases (1). This spectrum includes congenital hepatic fibrosis, polycystic liver disease, biliary hamartomas, and Caroli disease, which corresponds to Todani type V in the choledochal cyst classification system (2–4). In contrast, Todani type I–IV choledochal cysts may have different embryologic backgrounds and are not universally classified as ductal plate anomalies (2, 3). Caroli disease is defined by non-obstructive intrahepatic bile duct dilatation, and bile stasis predisposes patients to recurrent cholangitis, intrahepatic lithiasis, progressive fibrosis or cirrhosis, portal hypertension, and acquired extrahepatic portosystemic shunts (3, 5).

On ultrasonography, intrahepatic bile ducts may appear as cystic or tubular dilatations, sometimes with calcification, in affected dogs (6, 7). Computed tomography (CT) is crucial for confirming biliary communication and detecting associated lithiasis (3, 4, 8). In Caroli disease, CT may also demonstrate the characteristic “central dot sign,” which represents portal venous radicles surrounded by dilated intrahepatic bile ducts (8).

While Caroli disease is well described in human medicine, reports in veterinary literature are rare (9). Sporadic cases have been documented in young dogs presenting with intrahepatic ductal sacculation, icterus, cholangitis, or lithiasis (6, 7, 10), and a few cases have also been reported in cats (11, 12). However, detailed imaging descriptions in veterinary patients remain scarce, and ultrasonography may misinterpret cystic intrahepatic structures as gallbladder duplication or other anomalies, underscoring diagnostic challenges (12, 13).

This case report describes the ultrasonographic, computed tomographic, and histopathological features of intrahepatic saccular bile duct dilatation consistent with Caroli-like disease and concurrent extrahepatic biliary obstruction (EHBO) in a young small-breed dog. It underscores the diagnostic value of CT in differentiating this entity from gallbladder duplication anomalies and highlights the clinical importance of recognizing uncommon biliary malformations and their complications in small animals.

## 2 Case description

**Figure 1:**
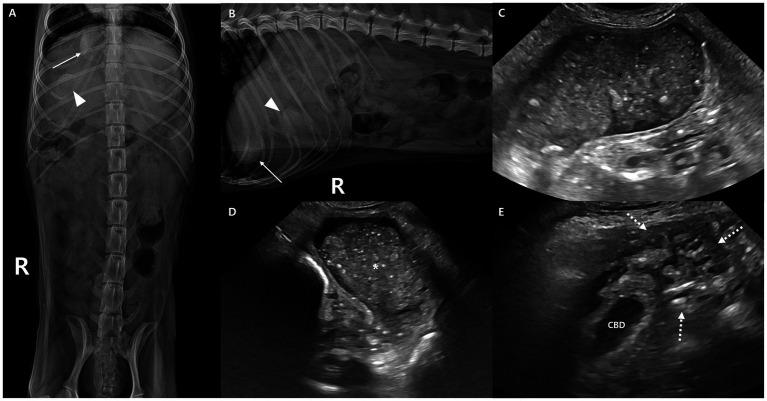
Abdominal radiographs and ultrasonography showing biliary disease. Ventrodorsal **(A)** and right lateral **(B)** radiographs reveal two distinct mineralized opacities (white arrow, white arrowhead) projected over the hepatic silhouette **(A,B)**. **(C)** Abdominal ultrasonography shows the gallbladder with intraluminal echogenic material (black asterisk) and a thickened wall. **(D)** A separate saccular structure (white asterisk) with similar intraluminal echogenicity is observed adjacent to the gallbladder. **(E)** Ultrasonography reveals marked dilatation of the common bile duct (CBD; diameter, 6.2 mm) and tortuous intrahepatic bile ducts (dotted arrow). Symbols: white arrow and white arrowhead = mineralized opacities projected over the hepatic silhouette on radiographs; black asterisk = gallbladder; white asterisk = adjacent cystic lesion.

**Figure 2:**
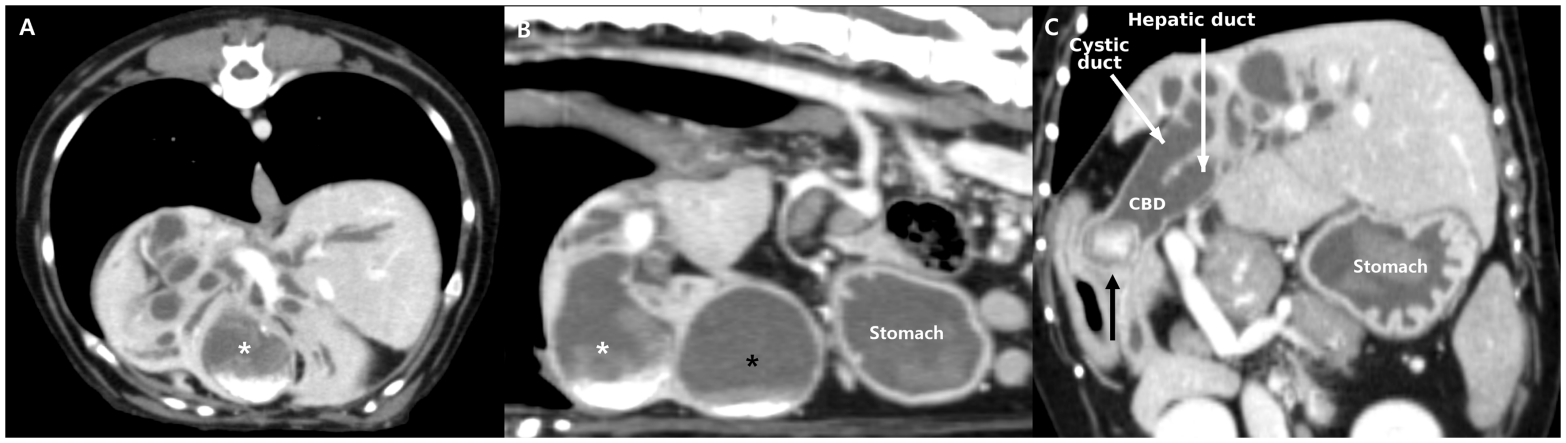
Computed tomography (CT) findings confirming saccular dilatation of the intrahepatic bile ducts and choledocholithiasis. **(A)** A post-contrast transverse image shows a well-circumscribed saccular cystic lesion (~2–3 cm, white asterisk) in the hepatic hilar region, containing heterogeneous hypoattenuating fluid and intraluminal mineralized material, accompanied by diffuse dilatation and tortuosity of the intrahepatic and extrahepatic bile ducts. **(B)** Sagittal reconstruction demonstrates the cystic lesion (white asterisk) separated from the gallbladder (black asterisk) and in continuity with the intrahepatic bile ducts. **(C)** Dorsal reconstruction highlights a dilated duct arising from the gallbladder, interpreted as the cystic duct, a dilated hepatic duct, and a markedly dilated common bile duct (CBD), measuring 8.1 mm, 9.0 mm, and 12.3 mm, respectively, together with a choledocholith (black arrow) at the major duodenal papilla, confirming extrahepatic biliary obstruction secondary to choledocholithiasis. Symbols: white asterisk = cystic lesion; black asterisk = gallbladder; black arrow = choledocholith.

We describe the case of a 2-year-old, 5.4 kg, castrated male small-breed dog (Maltipoo) who presented with a 2-month history of intermittent vomiting; weight loss, lethargy, and anorexia had been present for 1 month. The dog had no remarkable prior medical history, vaccination and deworming status were appropriate, and no known drug allergies were reported. On physical examination, the patient was depressed, moderately dehydrated, and markedly icteric. Hematology and serum biochemistry revealed severe hyperbilirubinemia (total bilirubin: 3.1 mg/dL; reference interval: 0.1–0.5 mg/dL); markedly increased liver enzyme activities (alanine aminotransferase [ALT]: 2,508 U/L [17–78 U/L], aspartate aminotransferase [AST]: 1,538 U/L [17–44 U/L], alkaline phosphatase [ALP]: >10,000 U/L [47–254 U/L], and gamma-glutamyl transferase [GGT]: 40 U/L [5–14 U/L]); and evidence of coagulopathy (prothrombin time [PT]: 20.1 s (14–19 s), activated partial thromboplastin time [aPTT]: 113.6 s (75–105 s), and elevated D-dimer 1,089 ng/mL [<250 ng/mL]). Abdominal radiographs showed two mineralized opacities projected over the hepatic silhouette, presumed to be associated with the gallbladder or the biliary tract (Figures 1A,B). Ultrasonography identified cholecystolithiasis, echogenic biliary sludge, and gallbladder wall thickening (Figure 1C). An additional cystic structure with intraluminal hyperechoic material, some of which produced distal acoustic shadowing, was observed adjacent to the gallbladder, raising concern for a duplicated gallbladder anomaly (Figure 1D). The common bile duct (CBD) and intrahepatic bile ducts were markedly dilated and tortuous, consistent with EHBO (Figure 1E). However, ultrasonography alone could not clarify the anatomical relationship of the cystic lesion to the biliary system, necessitating further evaluation with CT. CT demonstrated EHBO with a 1.3 × 1.8-cm choledocholith at the major duodenal papilla and multiple smaller calculi within the cystic and intrahepatic ducts, accompanied by diffuse dilatation and tortuosity of the biliary tree. A well-circumscribed saccular cystic lesion, measuring approximately 2–3 cm in diameter, was also identified in the hepatic hilar region, containing hypoattenuating fluid and intraluminal mineralized material. Multiplanar reconstructions showed that the cystic lesion was anatomically distinct from the gallbladder and formed part of the diffusely dilated biliary system, with direct continuity to the intrahepatic bile ducts. The lesion did not show the configuration of a separate accessory gallbladder-like reservoir with an independent cystic duct drainage pattern; these findings support the interpretation of saccular biliary dilatation within the Caroli-like disease spectrum rather than a duplicated gallbladder with two cystic ducts (Figure 2). In addition, heterogeneous enhancement of the left lateral lobe and papillary process was observed and interpreted as secondary hepatic parenchymal changes. Reactive enlargement of the hepatic and splenic lymph nodes was also observed. No concurrent renal cystic changes or other renal abnormalities were identified on ultrasonography or CT.

**Figure 3:**
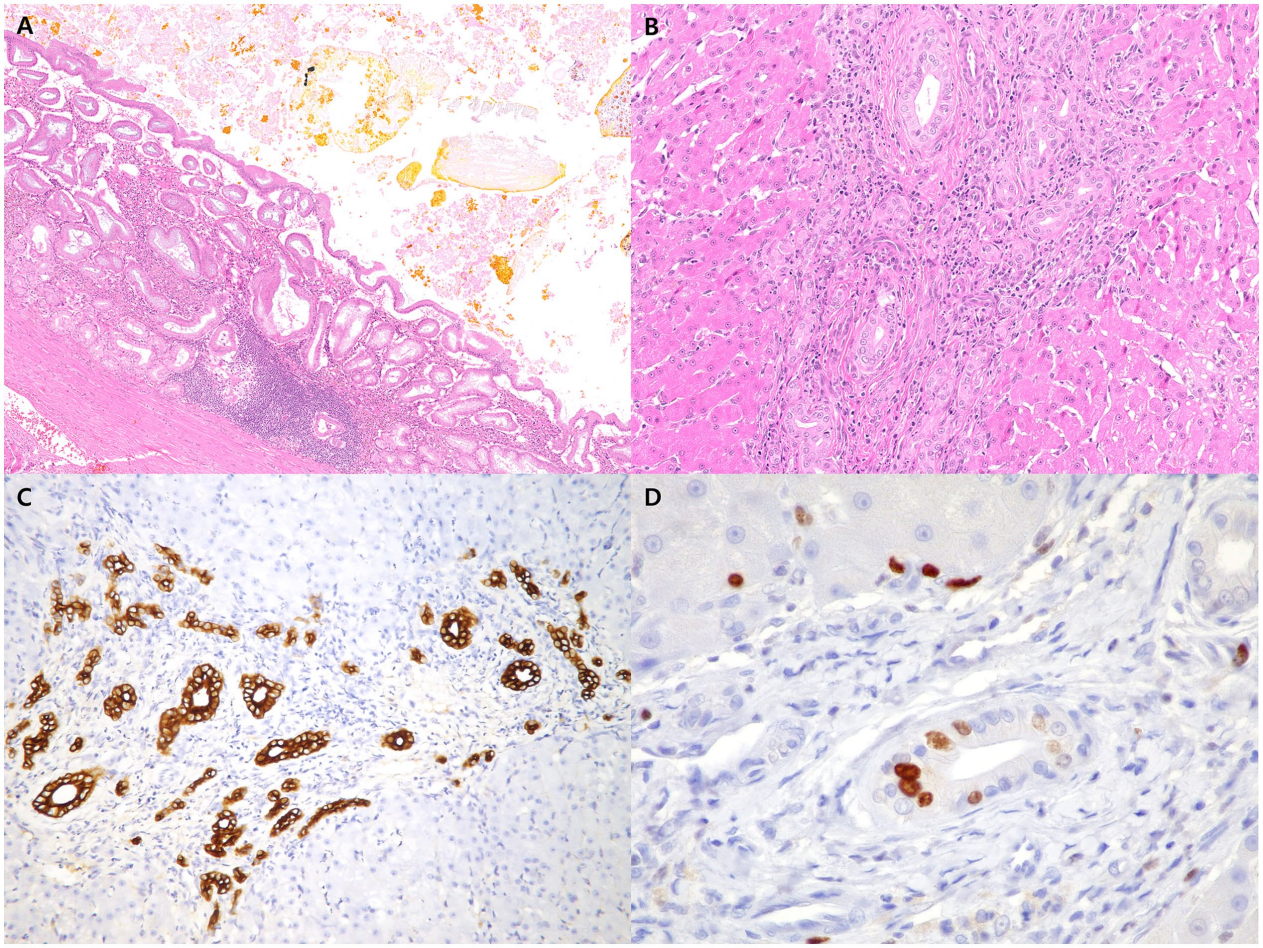
Histopathology and immunohistochemistry of the liver and choledochal cyst. **(A)** Choledochal cyst wall lined by tall columnar epithelium with mucinous hyperplasia and multifocal lymphoplasmacytic inflammation (H&E, ×10). **(B)** The liver parenchyma shows bridging cholangiohepatitis with periductal fibrosis and marked bile duct proliferation (H&E, ×20). **(C)** Immunohistochemistry confirms the biliary origin of the proliferating bile ducts, which exhibit strong cytoplasmic immunoreactivity for CK19 (CK19, ×20). **(D)** Rare Ki67-positive nuclei observed in bile ductules, indicating a low proliferation index (Ki67, ×40).

The patient underwent exploratory laparotomy and cholecystectomy. A duodenotomy was performed, and the choledocholith located at the level of the major duodenal papilla was removed. Retrograde flushing was then performed to confirm patency of the common bile duct. Bile was collected for microbiological culture, and tissue samples obtained from the liver and the wall of the cystic structure were submitted for histopathological evaluation. The sampled cystic structure corresponded to the same saccular lesion identified on ultrasonography and CT. *Escherichia coli* was isolated from the bile culture. Histopathological examination of the liver revealed moderate neutrophilic and lymphoplasmacytic bridging cholangiohepatitis with periductal fibrosis and marked bile ductule proliferation (Figure 3B). The cystic lesion wall was lined by tall columnar epithelium exhibiting mucinous hyperplasia with multifocal lymphoplasmacytic inflammation (Figure 3A). An immunohistochemical analysis revealed strong cytoplasmic immunoreactivity for cytokeratin 19 (CK19) in bile ductules, confirming their biliary epithelial origin (Figure 3C), whereas Ki67–positive nuclei were rare, indicating a low proliferative index (Figure 3D).

Taken together, these findings supported the diagnosis of a DPM with secondary inflammation, consistent with Caroli-like disease, corresponding to a Todani type V choledochal cyst. Despite surgical intervention, the patient’s condition deteriorated, and death occurred several days postoperatively. No necropsy was performed.

## 3 Discussion

Caroli disease, classified within the spectrum of DPM, is a well-characterized congenital anomaly in human medicine (1, 3) but remains rare in veterinary patients. Only a limited number of cases have been reported in dogs and cats (6, 7, 12), and the majority of them were described based on ultrasonographic or histopathologic findings with variable outcomes. The present case represents a young small-breed dog that developed intrahepatic saccular bile duct dilatation consistent with Caroli-like disease, complicated by concurrent EHBO and severe hepatobiliary inflammation. This constellation of findings highlights the dual challenges of diagnosing congenital biliary malformations in small animals and addressing their clinically significant sequelae.

A major diagnostic challenge in this case was the inherent limitation of histopathology in distinguishing a choledochal cyst from the gallbladder. Both structures share nearly identical wall architecture, composed of mucosa, connective tissue, vasculature, and inflammatory infiltrates, which makes them virtually indistinguishable under routine microscopy (4). For this reason, a combined interpretation of imaging and pathology is essential. In our patient, ultrasonography initially raised suspicion of a duplicated gallbladder because the cystic lesion contained echogenic material and appeared adjacent to the gallbladder (13, 14). However, CT with multiplanar reconstruction demonstrated direct continuity between the cystic lesion and the intrahepatic bile ducts, with no identifiable communication with the gallbladder. Based on this imaging pattern, Caroli-like disease, corresponding to a Todani type V choledochal cyst, was considered the most likely diagnosis (7, 8, 13, 15). An acquired biliary dilatation secondary to EHBO and cholangiohepatitis was also considered a differential diagnosis and could not be completely ruled out. EHBO typically produces diffuse tubular biliary dilatation; however, the marked saccular configuration of the lesion and its direct continuity with the intrahepatic bile ducts on CT were less typical of a purely acquired obstructive process. Thus, although the relative contribution of congenital DPM and secondary obstruction could not be fully resolved, the imaging findings supported Caroli-like disease as the most likely diagnosis. This emphasizes the decisive role of CT in avoiding misdiagnosis of biliary anomalies that cannot be reliably characterized by ultrasound or histology alone.

On CT, heterogeneous hepatic parenchymal enhancement indicated hepatocellular injury secondary to EHBO and cholangiohepatitis. These abnormalities corresponded well with histopathological evidence of bridging neutrophilic and lymphoplasmacytic cholangiohepatitis, periductal fibrosis, and marked bile duct proliferation. Gallbladder wall thickening with pericholecystic fat stranding was consistent with concurrent cholecystitis, whereas the presence of multiple calculi within both intrahepatic and extrahepatic ducts exemplified lithogenesis, a recognized complication of Caroli disease (12, 16). Moreover, *Escherichia coli* was isolated from the bile culture, consistent with chronic biliary stasis predisposing to stone formation and secondary bacterial cholangitis, as widely reported in human cases of Caroli disease (3, 17). Similar associations among biliary malformations, cholangitis, and bacterial infection have also been described in cats with suppurative cholangitis–cholangiohepatitis syndrome, in which ductal plate malformation was included among recognized comorbidities (18). Reactive enlargement of the hepatic and splenic lymph nodes further supported the presence of chronic inflammation. An immunohistochemical analysis revealed CK19-positive biliary epithelium with only rare Ki67-positive nuclei, indicating a low proliferative index. These findings are more consistent with congenital DPM than with post-injury ductal proliferation, although definitive differentiation remains limited. The young age of the patient (a 2-year-old dog) further supports a congenital origin, consistent with human reports of early-onset Caroli disease (1, 5). Similar immunohistochemical profiles have been described in canine DPM (19) and are well documented in human studies of Caroli disease and congenital hepatic fibrosis (4, 17). Taken together, these findings support the interpretation that congenital ductal plate malformation underlies Caroli-like disease in this patient, with chronic biliary stasis promoting lithogenesis and secondary infection, ultimately progressing to EHBO. Although the temporal sequence cannot be fully resolved by ultrasound and CT, this pathogenic cascade represents the most plausible explanation for the clinicopathological findings in this case report.

These correlations highlight the broad clinical implications of Caroli-like disease. Affected patients are predisposed not only to choledocholithiasis with subsequent EHBO and recurrent cholangitis but also to progressive hepatic fibrosis, portal hypertension, and eventual cirrhosis, as is well documented in human medicine (3, 5, 17). In veterinary patients, clinical manifestations such as vomiting, anorexia, jaundice, and weight loss are non-specific, which delays recognition and complicates management (6, 7, 12). This underscores the importance of CT imaging when ultrasonography reveals equivocal findings. In the present case, CT was decisive in demonstrating direct continuity between the cystic lesion and the intrahepatic ducts, making a duplicated gallbladder unlikely and supporting the diagnosis of Caroli-like disease corresponding to a Todani type V choledochal cyst (7, 8, 15).

In veterinary medicine, only a few cases of Caroli-like disease have been reported, including a CT-confirmed ductal communication in a crossbreed dog (7) and a case in a Rottweiler (10). Related hepatobiliary malformations have also been described in cats; necropsy/autopsy-based feline studies have reported hepatobiliary malformations and fibrocystic hepatobiliary lesions in approximately 13–14% of examined cases, with a substantial proportion interpreted as ductal plate malformations (12, 20). In contrast to the limited veterinary literature, Caroli disease is well characterized in human medicine as part of the DPM spectrum, with established complications such as intrahepatic lithiasis, recurrent cholangitis, and progressive fibrosis, as well as distinctive imaging features including the central dot sign (1, 3, 8), although this sign was not identified in the present case. This case contributes to the limited veterinary literature by providing CT-based confirmation of a cystic lesion’s biliary communication in a young small-breed dog, thereby linking veterinary findings with the broader human literature.

Despite surgical intervention, the prognosis was poor. The combination of EHBO, diffuse inflammatory changes, and lithiasis created a complex pathological environment that remained unresolved after surgery. *Escherichia coli* was isolated from the bile culture, underscoring the contribution of infection to disease progression. This adverse outcome parallels human cases, where recurrent cholangitis and progressive hepatic injury frequently determine long-term morbidity and mortality (3, 17).

A major limitation is that the histopathology cannot reliably distinguish a choledochal cyst from the gallbladder, as previously noted in human literature (1, 4). Liver immunohistochemistry with CK19 and Ki67 provided supportive evidence of ductular proliferation but is insufficient to establish a definitive diagnosis (19). This underscores the importance of CT in confirming biliary communication. Furthermore, as this is a single case, broader generalizability is inherently limited, and the absence of long-term follow-up further restricts interpretability. Nevertheless, the convergence of clinical, imaging, and pathological findings in this patient supports the diagnosis of Caroli-like disease, corresponding to a Todani type V choledochal cyst.

In conclusion, this case highlights the diagnostic pitfalls of relying on histology alone and demonstrates the essential role of CT in determining the precise anatomical localization of the lesion, particularly its communication with the biliary system, thereby preventing misclassification of biliary anomalies. To the best of our knowledge, it is the first CT-based case report of Caroli-like disease with concurrent EHBO in a young small-breed dog (Maltipoo).

## Data Availability

The original contributions presented in the study are included in the article/supplementary material, further inquiries can be directed to the corresponding author.
